# A surface topography analysis of the curling stone curl mechanism

**DOI:** 10.1038/s41598-018-26595-y

**Published:** 2018-05-25

**Authors:** Viktor Honkanen, Markus Ovaska, Mikko J. Alava, Lasse Laurson, Ari J. Tuononen

**Affiliations:** 1 Department of Applied Physics, Aalto University, P.O. Box 11100, FI-00076 Aalto, Espoo Finland; 2 Department of Mechanical Engineering, Aalto University, P.O. Box 14100, FI-00076 Aalto, Espoo Finland

## Abstract

The curling motion of the curling stone on ice is well-known: if a small clockwise rotational velocity is imposed to the stone when it is released, in addition to the linear propagation velocity, the stone will curl to the right. A similar curl to the left is obtained by counter-clockwise rotation. This effect is widely used in the game to reach spots behind the already thrown stones, and the rotation also causes the stone to propagate in a more predictable fashion. Here, we report on novel experimental results which support one of the proposed theories to account for the curling motion of the stone, known as the “scratch-guiding theory”. By directly scanning the ice surface with a white light interferometer before and after each slide, we observed cross-scratches caused by the leading and trailing parts of the circular contact band of the linearly moving and rotating stone. By analyzing these scratches and a typical curling stone trajectory, we show that during most of the slide, the transverse force responsible for the sideways displacement of the stone is linearly proportional to the angle between these cross-scratches.

## Introduction

**Figure 1:**
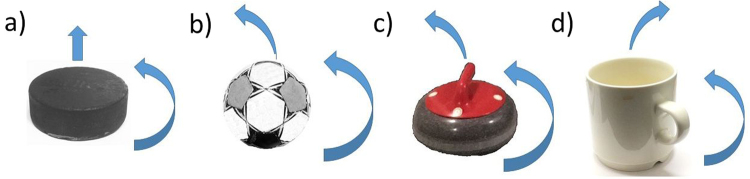
Different objects moving and rotating: (**a**) An ice hockey puck sliding on ice: rotation is not causing any lateral motion. (**b**) A football in the air: Magnus-effect is causing lateral displacement. (**c**) A curling stone on ice: similar lateral displacement to the football, but no consensus on the underlying physical phenomenon. (**d**) A coffee cup on an office table: moving to the right under counterclockwise rotation, possibly due to the load transfer to leading half because of high friction coefficient (deceleration) compared to ice.

The tendency of rotating and moving objects to travel along a curved trajectory is essential in many sports, and understanding the underlying physical mechanisms is a fascinating problem. Indeed, depending on the system, counter-clockwise rotation may lead to a curved trajectory towards left or right, or to no turn at all, see Fig. 1 for examples.

In the Olympic winter sport of curling, two teams slide granite stones across an ice sheet towards a target area. In each of the predetermined amount of game-parts (ends), points are awarded only to the team with a stone closest to the center of the target area: one point is scored for each stone beating the opposing team’s best stone. The essential characteristic of the game is a curled trajectory of the rotating curling stone and is well-known among the players: if a small clockwise rotational velocity is imposed to the stone when it is released, in addition to the linear propagation velocity, the stone will curl to the right. A similar curl to the left is obtained by counter-clockwise rotation. This effect is widely used in the game to reach spots behind the already thrown stones and rotation makes stone travel more predictable. What makes this phenomenon intriguing is that even if its existence is well-known, the underlying tribological mechanism remains to be explained properly. In fact, the mechanism that puts this curl in the curling stone is under scientific debate and novel experimental evidence is required.

The system of interest consists of the granite stone, weighing a little less than 20 kilograms, moving on the ice surface. The geometry of the stone is such that the apparent contact area between the stone and the ice surface is given by a circular band with a width of approx. 6 mm and a diameter of 120 mm. Both the bottom surface of the stone and the ice surface are rough: the bottom of the stone is intentionally roughened to control the magnitude of the ice-stone friction, as well as the curvature of the trajectory of the stone. The ice surface consists of many small protrusions known as pebbles, produced by spraying water droplets onto an initially flat ice surface. These pebbles, rough stone bottom surface, stone weight and small real contact area clearly form a combination favorable for a curled trajectory. As an example, the ice hockey puck does not have these properties and consequently it does not curl. and The stone is put to motion by releasing it before the release (hog) line, approx. 28 meters from the center of the target area. The player throwing the stone aims at adjusting the initial linear propagation velocity as well as the rotational velocity of the stone in order to get it to a desired final position within the target area. In addition, team mates may attempt to affect the motion of the stone by sweeping the ice in front of the sliding stone with special brooms. Under typical conditions of the game, with 3–4 revolutions during the propagation of the stone, the sideways displacement of the stone tends to be of the order of 1–1.5 meter.

**Figure 2:**
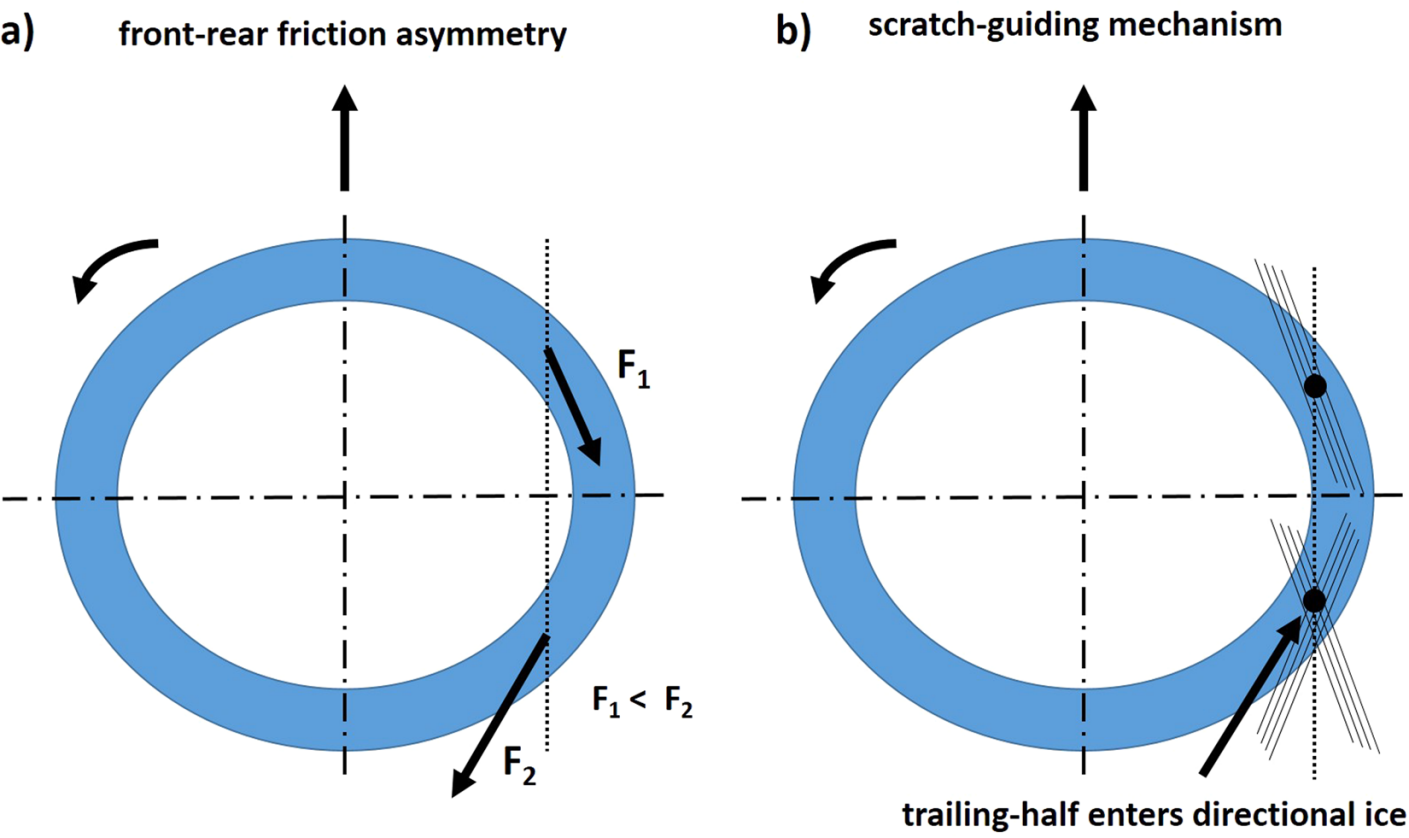
Previously proposed alternative mechanisms to explain the curled trajectory of a curling stone: (**a**) Front-rear friction asymmetry and (**b**) the scratch-guiding mechanism.

With the aim of explaining the empirically observed curved trajectory of the curling stone, several mechanisms have been proposed in the literature. One often-presented argument is related to a proposed asymmetry of the friction force between the leading and trailing parts of the contact band^1^. Indeed, if the leading half of the stone experiences a smaller friction force than the trailing half, the stone will be subject to a net lateral force towards right (left) in the case of clockwise (counter-clockwise) rotation, as illustrated in Fig. 2a. Notice that any asymmetry in friction between the left and right halves of the stone – as might arise due to a velocity-dependent friction law – cannot lead to a non-zero lateral net force: the different contributions of the lateral force along the circular band sum up to zero^2^. Mechanisms that have been proposed to lead to a front-rear asymmetry in friction include frictional melting of ice^1^, pickup of ice debris along the leading half of the contact band^3^, and temperature reduction originating from evaporation of a liquid film formed by the leading half^4^. However, the explanatory power of any such ideas is limited by the maximum lateral displacement they may account for: even in the extreme case of assigning all the friction to the trailing half, the resulting lateral displacement has been estimated to be approx. half of that empirically observed^5^.

Another recent theory trying to explain the phenomenon behind the curl of the curling stone is based on a “scratch-guiding” mechanism^6^. There, the idea is that the front half of the rough contact band scratches the ice, with the scratches tilted to the right or left from the propagation direction depending on the rotation direction of the stone (clockwise or counter-clockwise). The asperities of the trailing part of the rough contact band have to cross these scratches, generating another set of scratches, with the opposite tilt as compared to those due to the leading part (Fig. 2b). During this crossing, there would be a tendency of the leading part scratches to act as “guides” for the trailing part asperities, in analogy to the effect that should be familiar to anyone with experience in crossing tram tracks with a bicycle in an angle different from 90 degrees. This guiding mechanism would then provide a net force able to account for the observed lateral displacement^6^. However, even this idea has been recently criticized, partly because the supporting experimental evidence has been argued to be limited^7^. In particular, previously reported results obtained by studying resin replicas of the scratched pebbles suggest that only the scratches due to the trailing part of the stone would be observable^6^.

In this paper, we focus on observing the details of the topographical changes that occur on the surface of the ice sheet during the sliding of the curling stone. By directly scanning the ice surface in an almost *in situ* manner with a white light interferometer before and after each slide (travel), we observe cross-scratches caused by the leading and trailing edges of the linearly moving and rotating stone. Moreover, by analyzing the angle at which these scratches are formed for different combinations of translational and rotational velocity of the stone, we establish a strong correlation between the angular difference between the two sets of parallel scratches angle and the sideways displacement of the stone during a typical trajectory of the stone. Our observations and analysis thus supports the scratch guiding theory as the dominating mechanism explaining the curved trajectory of the curling stone.

## Results

To study the topographical features formed in the ice surface due to the sliding curling stone, we built a custom test track in a cold room (temperature around −5 °C). The track consists of an ice sheet with a removable part. This part of the ice sheet can be removed quickly from the track and placed in the white light interferometer for measurement immediately after sliding the curling stone over it, see Methods for details. This procedure allows us to directly scan the ice surface in an almost *in situ* manner, rather than studying a replica of the ice sheet as done in previous studies^6^.

**Figure 3:**
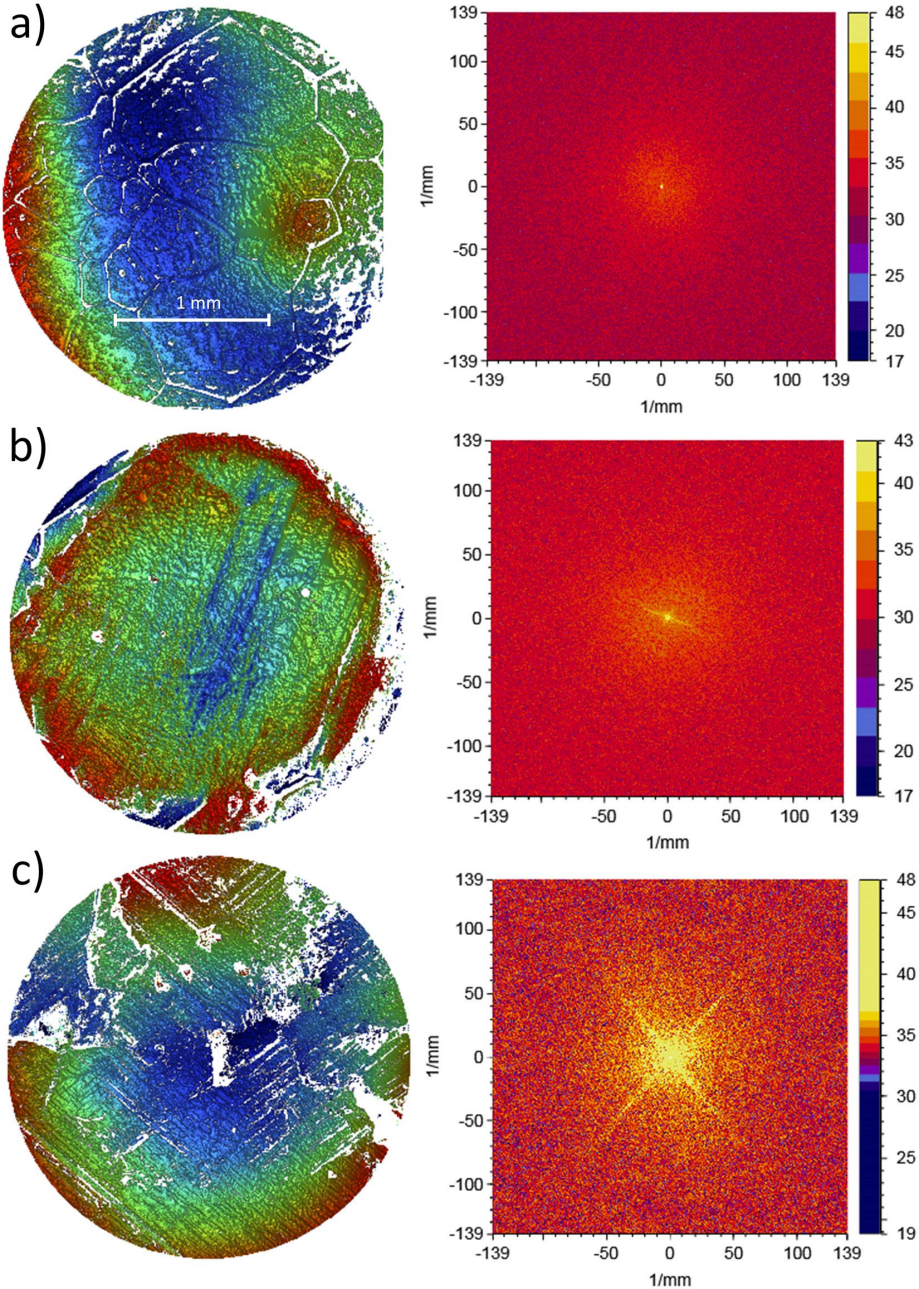
White light interferometry images of the surface topography of a pebble on the ice sheet. The curling stone slides over the pebble while exhibiting also clockwise rotation. (**a**) The surface topography before the slide (pristine pebble, left), together with the corresponding close-to-uniform PSD plot (right). (**b**) Same as in (**a**), but here the leading half of the curling stone has slid over the pebble. Notice the clear directionality of the scratch pattern, visible also in the PSD plot, corresponding to a single set of parallel scratches. (**c**) Both the leading and trailing halves of the contact annulus have slid over the pebble, resulting in two sets of parallel scratches.

Figure 3 shows examples of the surface profiles corresponding to pebbles before sliding (Fig. 3a, left panel), after only the leading half of the stone has slid over the pebble (Fig. 3b, left panel), and after both the leading and trailing halves have passed over the pebble (Fig. 3c, left panel). In all the cases shown, the sliding direction is “from down to up”. One can clearly observe a set of parallel scratches due to the leading half of the stone in Fig. 3b. Importantly, Fig. 3c displays quite clearly that after both the leading and trailing parts of the stone have slid over a pebble, two different sets of parallel scratches can be observed: one due to the leading and other due to the trailing half of the stone. The same conclusion can be reached by considering the power spectral density (PSD) plots of the surface profiles, quantifying the strength of the variations as a function of frequency. For example, if there are scratches in one direction, then the PSD plot exhibits a strong line in the direction perpendicular to the scratches. The PSD plots corresponding to the surface profiles are shown in the right panels of Fig. 3a–c. While the pristine pebble exhibits a close-to-isotropic PSD (Fig. 3a, with some weak structure visible in the PSD due to grain boundaries), clear signatures of one (after only the leading edge has scratched the surface, Fig. 3b) and two (when scratches due to both the leading and trailing edges are present, Fig. 3c) sets of parallel scratches are observable.

**Figure 4:**
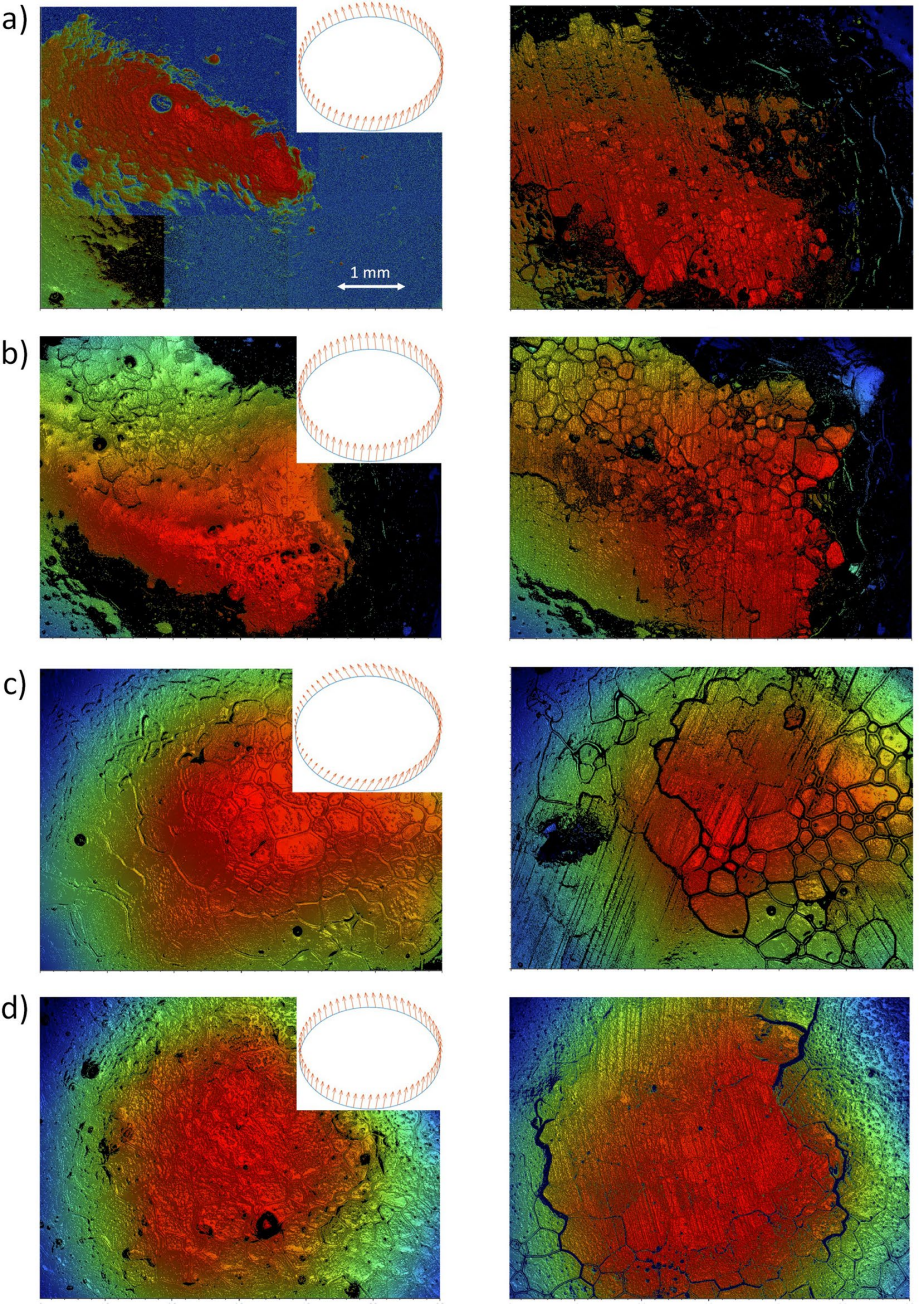
Surface topography images of a pebble in the ice sheet, corresponding to four slides with different translational (upwards) and rotational speeds (counterclockwise). (**a**) Fast sliding (~1 m/s) and fast rotation (~4.5 rad/s). (**b**) Fast sliding and slow rotation (~1.3 rad/s). (**c**) Slow sliding (~0.5 m/s) and fast rotation. (**d**) Slow sliding and slow rotation. The topography before the slide is shown on the left, while the corresponding topography after the slide is shown on the right. Vector plots represent the velocity vectors of each point of the contact annulus; these correspond well to the observed orientation of the two sets of parallel scratches.

Moreover, Fig. 4 shows four examples of surface profiles before and after the slide, considering four different combinations of translational and counterclockwise rotational speeds (“low”, about 0.5 m/s and 1.3 rad/s, and “high”, about 1 m/s and 4.5 rad/s, thus giving rise to four different situations); in each case, the left panels show the pebble before the slide, while the right panels show the same pebbles after the stone has slid over them. For reference, we also show speed vectors of each point of the contact annulus. For most cases, one can observe two sets of scratches, one due to the leading and the other due to the trailing edge. The orientation of these scratches is consistent with the speed vectors of the leading and trailing edges: for instance, the angle between the scratches and the propagation direction of the stone (from down to up) is the largest in Fig. 4c, corresponding to the “slow sliding and fast rotation case”, while the opposite case of “fast sliding and slow rotation” (Fig. 4b) leads to scratches that are almost parallel to the sliding direction. Thus, we conclude that these surface profiles obtained from our white light interferometer measurements clearly show that in a typical case, the sliding of the curling stone over a pebble results in two sets of parallel scratches with their orientations given by the velocities of the leading and trailing edges of the contact annulus.

**Figure 5:**
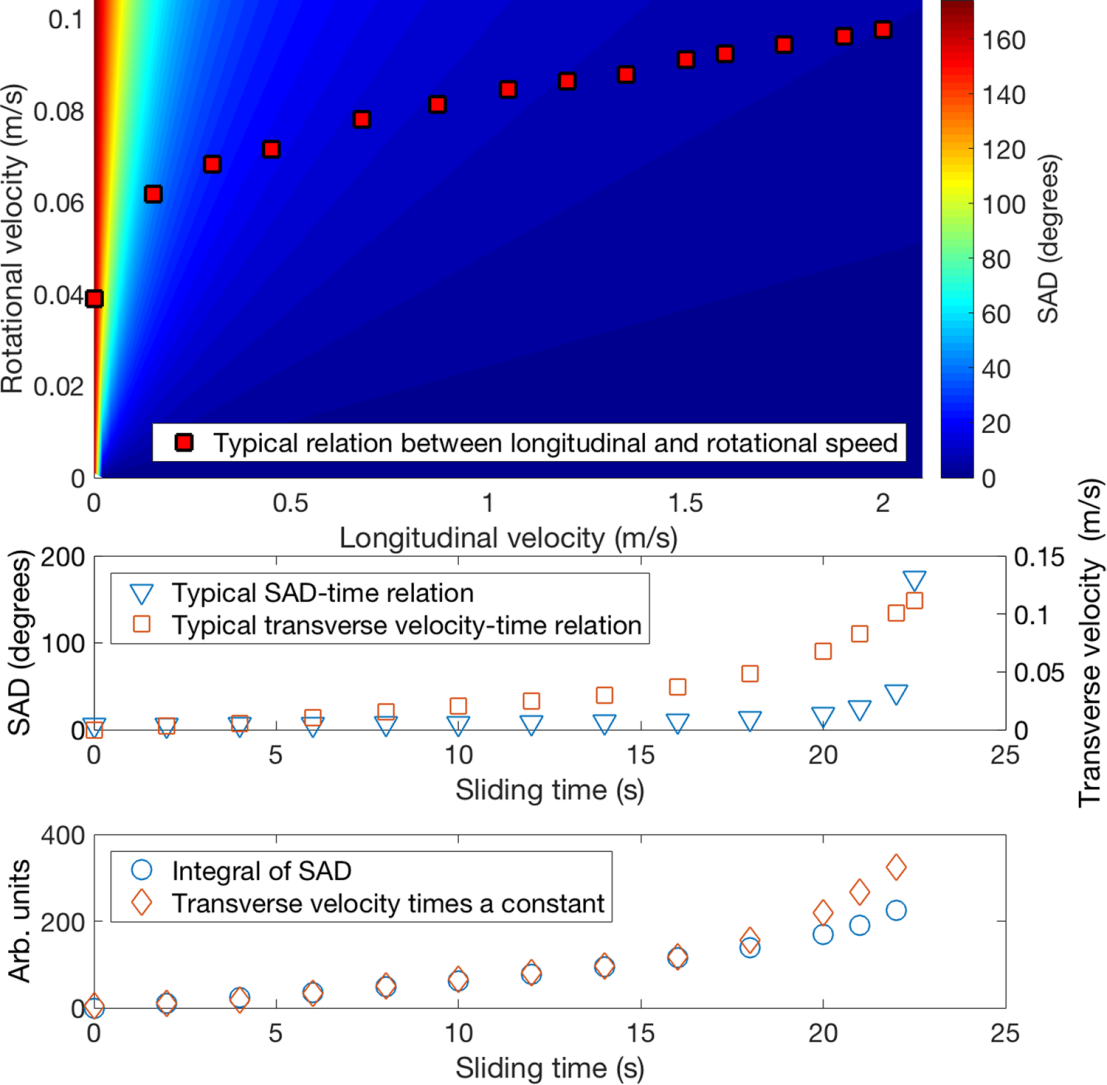
Top: The color map shows the angle between the front-most and the rear-most speed vectors as a function of the rotational speed of the contact annulus and the translational speed of the center of mass of the stone; this quantity is taken to be a proxy of SAD. A typical relation between the longitudinal and rotational speeds (data obtained from ref.^11^) is superimposed. Middle: Typical SAD-time relation and typical transverse velocity-time relation (data obtained from ref.^11^), indicating that some correlation between the curl and SAD exists. Bottom: Comparison of the time integral of SAD and the transverse velocity, showing that the two are linearly proportional [as predicted by Eq. (2)] up to sliding time of roughly 18 s, with only the last few seconds of sliding, corresponding to large SAD values, displaying deviation from the prediction of the linearized model.

Finally, we consider the typical time-evolution of the longitudinal and rotational velocities, and hence of the angle between the velocity vectors of the leading and trailing edges of the contact annulus during the typical dynamics of the curling stone; the data analyzed is obtained from ref.^4^. As shown above, the angle between the velocity vectors of the leading and trailing edges equals the angle between the two sets of scratches observed in white light interferometry measurements; in what follows, we’ll refer to this angle difference as the *scratch angle difference* (SAD). Initially, as the longitudinal velocity dominates, SAD is small, see Fig. 5. As the propagation velocity of the stone slows down, SAD starts to increase, and, at the very end of the sliding motion, increases quite rapidly towards 180°, corresponding to pure rotational motion. Indeed, as observable in the top panel of Fig. 5, the rotational velocity still has a small but finite value even when the longitudinal velocity has gone to zero; regarding this point, our results thus seem to contradict those of ref.^8^, where the sliding and spinning degrees of freedom were found to be coupled. In the middle panel of Fig. 5, we compare the time-evolution of the transverse velocity of the stone and that of SAD (in middle of the curling stone). It is evident that some correlation between the curl of the curling stone and SAD exists.

The general SAD dependence of the transverse force responsible for the curved trajectory of the stone originating from the scratch-guiding mechanism is difficult to establish or derive, due to the complexity of the underlying microscopic processes. However, one may argue that the transverse force *F*_trans_ should be some function *F*_trans_(*θ*) of SAD, denoted here by *θ*. For small *θ*, one may linearize *F*_trans_(*θ*), and thus arrive at an equation of motion describing the transversal motion of the stone for small *θ*,

1 $$M\frac{{\rm{d}}{v}_{{\rm{trans}}}}{{\rm{d}}t}=C\theta ,$$

where *M* is the mass of the stone, and *C* a constant. Integrating Eq. (1) then leads to

2 $${v}_{{\rm{trans}}}(t)=\frac{C}{M}\,{\int }_{0}^{t}\,\theta (t^{\prime} ){\rm{d}}t^{\prime} ,$$

i.e., *v*_trans_(*t*) is proportional to the time integral of *θ*. In the bottom panel of Fig. 5, we compare the time integral of SAD to the transverse velocity. Rescaling the latter with a suitable constant leads to a nice collapse of the data with the exception of the last few data points which correspond to high values of SAD such that the linearization of *F*_trans_(*θ*) employed above breaks down. The linearization seems to work well for SAD-values smaller than *θ* ≈ 25°, corresponding to the dark blue region in the top panel of Fig. 5. One may notice that in this regime also the relation between the rotational and longitudinal speeds is roughly linear (see the data points in the top panel of Fig. 5). During the last few seconds of sliding, non-linear effects not captured by the above linearized description start to become important.

## Conclusions

In this paper we have presented the first experimental, nearly *in situ* observation of cross-scratches created by the leading and trailing halves of the contact annulus of the curling stone. The orientation of these scratches is given by the local velocity vectors along the contact annulus of the propagating and rotating stone. The presence of these two sets of parallel scratches allows one to define the quantity we refer to as the scratch angle difference (SAD), which depends on the relative magnitudes of the longitudinal and rotational velocities of the stone. We found that the transverse force responsible for the curved trajectory is linearly proportional to SAD (denoted by *θ*) for small *θ* (*θ* < 25° or so), and hence the time-dependence of the transverse velocity is linearly proportional to the time integral of *θ*. Together, these two observations provide strong evidence that at least up to a few seconds before the stone comes to a halt, the dominating contribution responsible for the curl of the curling stone is given by the scratch-guiding mechanism^6^.

For large SAD, i.e., typically during the last few seconds of the slide of the stone, the above-discussed linearization breaks down. This could be due to two main reasons: (i) the dependence of the transverse force on SAD, *F*_trans_(*θ*), may become non-linear, so that the dominating contribution to *F*_trans_ would still be due to the scratch-guiding mechanism, or (ii) some other mechanism may start operating. For instance, one could argue that the scratch-guiding mechanism may become stronger for small longitudinal velocities because of different fracture behaviors operating for different time scales (“more time to follow scratches”), thus giving rise to the strongly curved trajectory at the very end of the slide. On the other hand, one could imagine other mechanisms that might play a role, including for instance possible local sticking of the “small sliding velocity” quarter of the contact annulus. Obviously, several different mechanisms may be operating simultaneously. In any case, the final stages of the sliding process appear to be governed by complex, non-linear mechanisms originating from microscale contact mechanics. Thus, understanding the related details poses an important challenge to be tackled in future work.

## Methods

### Experiments

**Figure 6:**
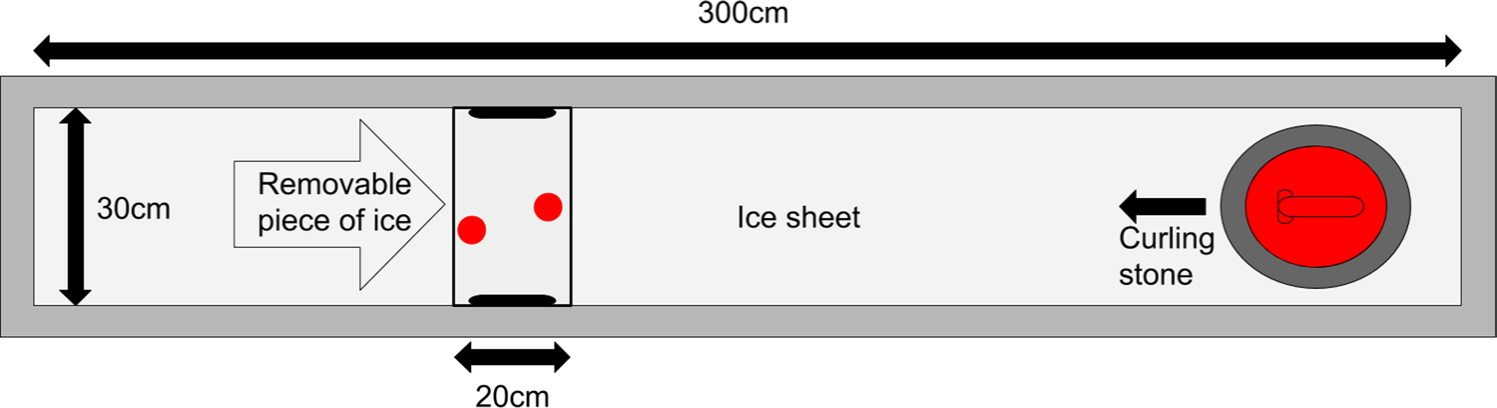
A sketch of the experimental setup. The red dots on the removable part of the ice sheet show roughly the positions of the pebbles scanned with the white light interferometer.

The experimental setup was built in a cold room (temperature around −5 °C and relative humidity 80–85%). A sketch of the setup is shown in Fig. 6.

Three sheets of plywood were put on a base with metallic edges to construct the basis of the ice sheet. The smallest plywood sheet (length: 20 cm) was removable and positioned as indicated in Fig. 6. Tap water was used for freezing the ice sheet. After the ice sheet was thick enough a spray bottle was used to make the top layer pebbled.

For scanning the surface of the ice sheet a white light interferometer (Bruker Contour GT-K Automated System), which was also placed in the cold room, was used. The same experimental methodology has been previously used to study rubber-ice friction^9^ and ice skate friction^10^. A plate was installed in the interferometer in order to attach the removable piece of ice exactly in the same position in the different experiments. The spatial resolution of the interferometer is 55 nm, but accuracy is restricted by the scanning light wavelength. Images of a width of 6 mm and length of 4,5 mm were scanned before and after each slide. After each slide the sheet was repaired by spraying water on it.

Different sliding configurations were used to mimic diverse scenarios: We considered rotating the stone without propagation, rotating clockwise and counterclockwise, slides with different combinations of translational and rotational speeds, as well as slides in which either only the front edge or the whole stone crossed the pebble.

### Image data analysis

**Figure 7:**
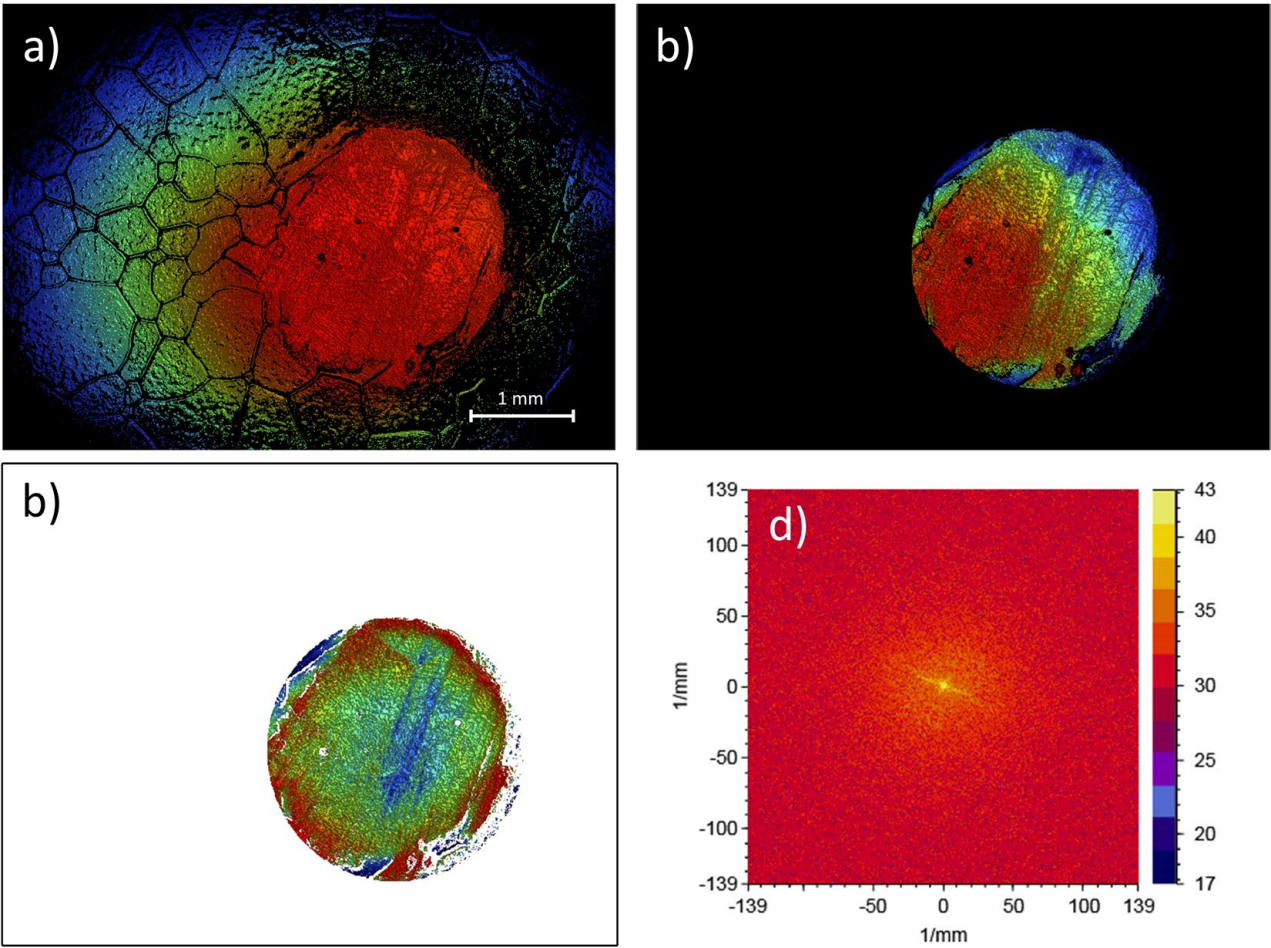
Data analysis process: (**a**) Raw topography of the pebble, (**b**) masked data, (**c**) data with tilt and 61.7 mm sphere removed from masked data, (**d**) 2D power spectral density (PSD) showing directionality of the surface structure.

The directionality of surface topography was shown by two-dimensional PSD plots in Fig. 3. The analysis process is illustrated in Fig. 7. In the original topography, directionality is dominated by randomly oriented ice grain boundaries that are not in contact to the curling stone. Thus, the topography around the pebble was masked leaving only curved top profile of the pebble for further analysis. In order to study pure scratch directionality on top of the surface, the tilt of the image and 61.7 mm sphere (analyzed pebble curvature) was subtracted from the image. After these consecutive steps, the resulting image contains only information about the surface scratches and 2D PSD plots are a convenient method to illustrate them.

### Data availability

The datasets generated during and analyzed during the current study are available from the corresponding author on reasonable request.
